# Non-Targeted Serum Lipidomics Analysis and Potential Biomarkers of Laryngeal Cancer Based on UHPLC-QTOF-MS

**DOI:** 10.3390/metabo12111087

**Published:** 2022-11-09

**Authors:** Haoyue Wang, Yanbo Luo, Huan Chen, Hongwei Hou, Qingyuan Hu, Min Ji

**Affiliations:** 1 Anhui Institute of Optics and Fine Mechanics, Hefei Institutes of Physical Science, Chinese Academy of Sciences, Hefei 230031, China; 2 Science Island Branch, Graduate School of University of Science and Technology of China, Hefei 230026, China; 3 Key Laboratory of Tobacco Biological Effects, China National Tobacco Quality Supervision & Test Center, Zhengzhou 450001, China

**Keywords:** laryngeal cancer, lipidomics, lipid metabolism, biomarkers, liquid chromatography–tandem mass spectrometry, serum

## Abstract

Laryngeal cancer is a common head and neck malignant cancer type. However, effective biomarkers for diagnosis are lacking and pathogenesis is unclear. Lipidomics is a powerful tool for identifying biomarkers and explaining disease mechanisms. Hence, in this study, non-targeted lipidomics based on ultra-performance liquid chromatography–quadrupole time of flight–mass spectrometry (UHPLC-QTOF-MS) were applied to screen the differential lipid metabolites in serum and allowed for exploration of the remodeled lipid metabolism of laryngeal cancer, laryngeal benign tumor patients, and healthy crowds. Multivariate analysis and univariate analysis were combined to screen for differential lipid metabolites among the three groups. The results showed that, across a total of 57 lipid metabolic markers that were screened, the regulation of the lipid metabolism network occurred mainly in phosphatidylcholine (PC), lysophosphatidylcholine (LPC), and sphingomyelin (SM) metabolism. Of note, the concentration levels of sphingolipids 42:2 (SM 42:2) and sphingolipids 42:3 (SM 42:3) correlated with laryngeal cancer progression and were both significantly different among the three groups. Both of them could be considered as potential biomarkers for diagnosis and indicators for monitoring the progression of laryngeal cancer. From the perspective of lipidomics, this study not only revealed the regulatory changes in the lipid metabolism network, but also provided a new possibility for screening biomarkers in laryngeal cancer.

## 1. Introduction

Laryngeal cancer is the second most prevalent respiratory cancer type after lung cancer. The World Cancer Report 2020 showed that the numbers incidence and mortality are estimated to be 184,600 and 99,800 worldwide, respectively, which is an increase of 4.1% and 5.2% compared to the results reported in 2018 [1,2]. About 60% of patients with laryngeal cancer were in stage III or IV at first diagnosis [3], while the 10-year survival rate of patients with stage I and II cancer who received radiotherapy or surgery were 90% and 70%, respectively [4]. The gold standard for predicting laryngeal lesions is laryngoscopy plus histopathology, which is invasive and expensive. In recent years, squamous-cell-carcinoma-associated antigen (SCCAg), cytokeratins 21-1 (CYFRA 21-1), carbohydrate antigen 72-4 (CA72-4), and carbohydrate antigen 199 (CA199) have been considered as tumor biomarkers in the initial diagnosis of laryngeal cancer [5,6]. However, these proteins have low specificity because they are aberrantly expressed in other cancer types such as lung and cervical cancers [7,8]. Therefore, there is an urgent need to explore effective biomarkers of laryngeal cancer.

During the process of cancer development, metabolic reprogramming causes changes in the levels of metabolites including amino acids, nucleic acids, lipids, and polysaccharides [9]. In particular, lipids are biologically important and disease-related because they not only form membrane structures and store energy, but are also active partners of transmembrane proteins and regulators of tumor growth as signaling molecules [10]. De et al. and Li et al. demonstrated, using in vitro experiments, that fatty acid synthase (FASN) and acetyl-CoA carboxylase (ACC) are highly expressed in laryngeal cancer tissues to promote fatty acid synthesis [11,12]. The level of phosphatase and tensin homolog deleted on chromosome ten (PTEN) is down-regulated in laryngeal cancer, a gene involved in regulating lipid metabolism by affecting Akt/mTOR signaling, FASN and ACC expression, and lipogenesis [13]. According to these results, laryngeal cancer pathogenesis involves lipid metabolism dysfunction caused by lipid-related genes and enzymes being abnormally expressed. In order to further understand the pathogenesis of laryngeal cancer, comprehensive studies of lipid molecules should be undertaken.

Lipidomics is a branch of metabolomics, which provides a comprehensive interpretation of lipid metabolism changes in disease, marker discovery, and mechanism explanation. As a type of biochemical indicator, lipids are more closely related to a phenotype than enzyme or protein biomarkers and have the advantage of being harvested noninvasively and sensitively. In the present study, serum lipidomics analysis was performed based on UHPLC-QTOF-MS in laryngeal cancer patients (LC), laryngeal benign tumor patients (LBT), and healthy crowds (HC). Principal component analysis (PCA), orthogonal partial least squares discriminant analysis (OPLS-DA) of multivariate analysis, and combining the Mann–Whitney U test as one of the nonparametric tests of univariate analysis were used to screen for differential lipid metabolites among three groups. Collectively, this study screened differential lipids, explored the characteristics and biological significance of lipid metabolic remodeling in laryngeal cancer, and provided new possibilities for screening potential biomarkers of laryngeal cancer.

## 2. Materials and Methods

### 2.1. Chemical and Reagents

Ammonium formate (purity > 99.99%), sodium hydroxide (purity > 98.0%), and formic acid of chromatographic grade (purity > 98.0%) were obtained from Sigma-Aldrich (Sigma-Aldrich, Beijing, China). HPLC grade acetonitrile and isopropanol were supplied by Merck (Merck KGaA, Darmstadt, Germany). Ultrapure water was prepared with a Milli-Q system (Millipore, Bedford, OH, USA).

### 2.2. Serum Collection

A total of 30 serum samples from LC, LBT, and HC conditions were obtained from the Tumor Hospital affiliated with Zhengzhou University and Zhengzhou Central Hospital, affiliated with Zhengzhou University. This study has been approved by the ethics committee of the School of Basic Medical Sciences, Zhengzhou University (approval number 18), and each participant signed informed consent. The sample information of all subjects is shown in Table 1. Subjects with cardiovascular, liver, kidney, or blood system dysfunction were excluded. Multiple aliquots for each subject were collected and stored at −80 °C for further analysis.

### 2.3. Sample Preparation

Serum samples were thawed at 4 °C for 2 h. An amount of 120 μL of isopropanol was added to triplicate serum samples (40 μL each), then mixtures were vortexed and incubated at room temperature for 10 min. The mixtures were stored at −20 °C overnight and centrifuged for 20 min (14,000× *g*, 4 °C) on the following day. An amount of 100 μL of the supernatant was collected carefully and 100 μL of acetonitrile: water (2:98, *v*/*v*) solution was added to adjust the water content to 50% for subsequent analysis. The analytical sequence consisted of quality control (QC) samples and triplicate analysis of each serum sample. QC samples were prepared from five pooled samples from each of the three groups in the same volume and were used for the evaluation of the stability of the analysis system during lipidomics analysis. The pretreatment of QC samples was the same as for the subject samples.

### 2.4. Lipidomics Analysis Based on UHPLC-QTOF MS Platform

The non-targeted lipidomics analyses were performed on a DIONEX Ultimate 3000 UHPLC system (Thermo Fisher Scientific, Waltham, MA, USA) coupled with an Impact II quadrupole time-of-flight mass spectrometer (Bruker Daltonics Corporation, Karlsruhe, Germany). A volume of 2 μL of each sample was injected by the autosampler. The column used was an ACQUITY UPLC CSH C18 (2.1 × 100 mm, 1.7 μm), with a flow rate of 0.4 mL/min and column temperature of 55 °C. Mobile phases consisted of A (10 mM ammonium acetate and 0.1% formic acid in water/acetonitrile = 40/60, *v*/*v*)-B (10 mM ammonium acetate and 0.1% formic acid in isopropanol/acetonitrile = 90/10, *v*/*v*). The liquid chromatography gradient was as follows: 0–2.0 min solvent B began from 40% to 43% and rapidly increased to 50% in 0.1 min, then linearly increased to 54% B at 12.0 min and further increased to 70% B in 0.1 min. Between 12.1 and 18.0 min, solvent B linearly increased to 99%, then was reduced to 40% B in 0.1 min and held for 1.9 min post-equilibrium. The UHPLC-QTOF-MS lipidomics data acquisition was performed in electrospray positive ion (ESI+) scan mode. The detailed MS parameters were set as follows: spray voltages, 4.5 kV; capillary temperature, 220 °C; dry gas flow rate, 8 L/min; nebulizer pressure, 1.8 bar; collision energy, 7 eV; transfer time, 80 μs. The MS/MS information of lipid metabolites was obtained by collision-induced dissociation experiments and data dependence acquisition (DDA). The mass scan range was 50–1300 Da and the scanning frequency was 10 Hz. The mass accuracy was corrected using sodium formate solution (10 mmol/L) as a calibration standard. At the beginning of each analysis batch, six QC samples were injected, and QC samples were re-analyzed after every nine injections of the subject samples. Ten samples of each group were performed to screen significant lipid differences in LC and LBT compared with HC and to explore the serum lipid metabolic signatures of LC.

### 2.5. Data Processing

After obtaining the raw lipidomics data of all samples, the data were processed by Metaboscape^®^ 4.0 (Bruker Daltonics Corporation, Karlsruhe, Germany). The raw peak values of all outputs were exported to Microsoft Excel to obtain peak tables. All sample data were corrected by MetaboAnalyst (http://www.metaboanalyst.ca (accessed on 20 February 2022)) for missing value estimation, data scaling, and normalization. The identification of lipids is annotated by a home database of Metaboscape^®^ 4.0 software, which can be queried according to the spectral library of accurate molecular weight, characteristic peak information, MS/MS spectrum data, and adducts. The information, including error mass, isotope patterns, and retention time, can be provided to quickly obtain qualitative lipid results.

### 2.6. Statistical Analysis

Statistical analysis was performed based on adjusted peak tables. The data matrix was imported into SIMCA-P 14.0 (Umetrics, Umea, Sweden) for multivariate statistical analysis. The clustering degree of QC samples was performed by principal component analysis (PCA) to evaluate the stability of the analysis system across different batches. Samples of three groups under the PCA model separation revealed a significant difference between groups. An orthogonal partial least-squares-discriminant analysis (OPLS-DA) was used to screen variables with variable importance in the projection (VIP) > 1. A permutation test was exploited to verify the fitting degree of the OPLS-DA model. R2 represents the explanation capacity of the model, while Q2 represents the predictive capacity of the model. When R2 < 0.4 and Q2 < 0.05, the built model is not overfitting. Univariate analysis was analyzed using SPSS 22.0 (IBM, Chicago, IL, USA) software. Lipids with P < 0.05 and VIP > 1.0 in the OPLS-DA were screened as differential metabolites by the non-parametric Mann–Whitney U test of the Student’s t-test.

## 3. Results and Discussion

### 3.1. Non-Targeted Lipidomics Profiling Analysis and Quality Control

**Figure 1:**
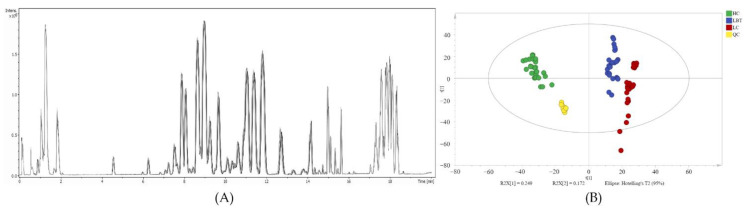
Total ion current chromatograms of QC serum samples (n = 15) (**A**), and principal component analysis (PCA) results of LC, LBT, and HC (**B**). (HC, healthy crowds; LBT, laryngeal benign tumor patients; LC, laryngeal cancer patients; QC, quality control.)

The total ion flow plots of the QC samples were superimposed, illustrating that the peak intensity and retention time of each chromatograph substantially overlapped. The results indicated that the batch effect was small, and the stability of the instrument was good during analysis (Figure 1A). The peak values were normalized by unit variance scaling. In the scoring plot of the PCA model (Figure 1B), QC samples were well clustered, indicating that the stability and repeatability of the instrument and process were satisfactory. The classification trend among the samples of LC, LBT, and HC is obvious, indicating the presence of biomarkers that may be identified (Figure 1B).

### 3.2. Significant Lipid Differences in LC and LBT Compared with HC

After data normalization, to identify a molecular-signature-based classification, an integrated multivariate analysis of 30 samples in three groups was conducted. Under the PCA model, samples from LC, LBT, and HC were obviously separated, confirming that variables observed in the sample were non-systematic and were, instead, biologically relevant. There were some cross-overlapping samples between the LC and LBT groups, which may relate to the degree of progression of benign tumors.

**Figure 2:**
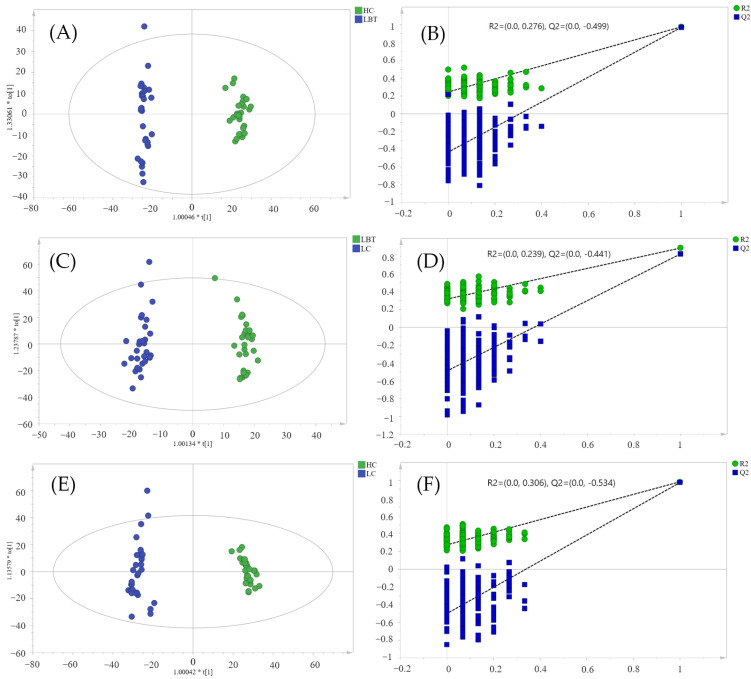
Orthogonal partial least-squares-discrimination analysis (OPLS-DA) and permutation test results of LC, LBT, and HC (LC, laryngeal cancer patients; LBT, laryngeal benign tumor patients; HC, healthy crowds). OPLS-DA model of HC and LBT (**A**), LBT and LC (**C**), HC and LC (**E**). Permutation test results of HC and LBT (**B**), LBT and LC (**D**), HC and LC (**F**).

OPLS-DA models were outlined to summarize the differences in serum lipid metabolic profiling between any two means. In the OPLS-DA models of LC and HC (Figure 2A,B), LBT and HC (Figure 2C,D), and LC and LBT (Figure 2E,F), the R2Y values were 0.988, 0.991, and 0.981, respectively, and the Q2 values were 0.981, 0.986, and 0.920, respectively. All of these values were close to 1, indicating a strong predictive ability of the model. The results of 200 permutation tests indicate that the three OPLS-DA models created were not overfitting and the model is reliable. Based on the above models, differential lipid metabolites passing the VIP threshold (VIP > 1) were considered to contribute to the distinction between the two groups.

**Figure 3:**
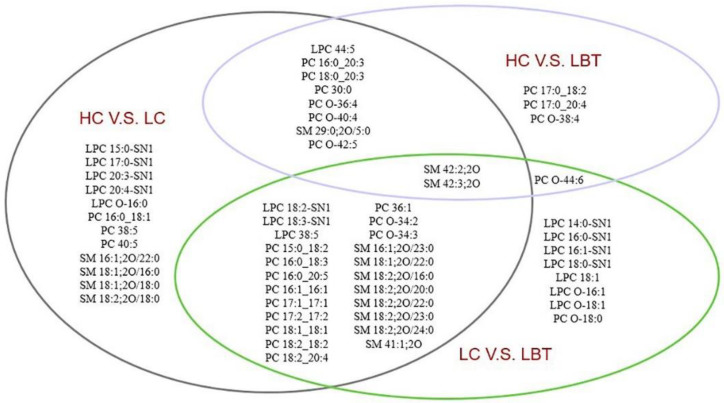
Significantly different lipids from across LC, LBT, and HC groups (LC, laryngeal cancer patients; LBT, laryngeal benign tumor patients; HC, healthy crowds).

Univariate analysis was performed using SPSS 22.0 software, and non-parametric tests (Mann–Whitney U test) were used to screen out variables meeting the criterion of P < 0.05 based on significant differences. The combination of univariate and multivariate analysis was able to effectively screen out differential lipids, and a total of 57 lipids met VIP >1 and P < 0.05 and were thus screened out (Figure 3). The qualitative information from the 57 differential lipids is shown in Table S1. There were 45 differential lipids in the healthy group compared with the cancer group, 14 differential lipids in the HC group compared with the LBT group, and 34 differential lipids in the LC group compared with the LBT group. By Venn diagram analysis, the group differences in lipid metabolism were more significant in the comparisons between LC and HC, and between LC and LBT. This is likely because cells in laryngeal benign tumors are not malignant, meaning that their lipid metabolism is more similar to healthy cells as opposed to cancer cells.

**Figure 4:**
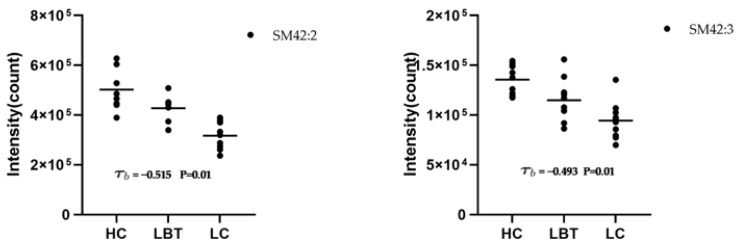
Mean plot of SM 42:2, SM42:3 among LC, LBT, and HC groups (SM 42:2: sphingomyelin 42:2; SM 42:3, sphingomyelin 42:3; LC, laryngeal cancer patients; LBT, laryngeal benign tumor patients; HC, healthy crowds).

In addition, sphingomyelin 42:2 (SM 42:2) and sphingomyelin 42:3 (SM 42:3) were observed at significantly different levels between any two groups, implying that they were present at different concentrations in three groups and might be biologically relevant in the disease process (Figure 4). Bivariate correlation analysis was used to evaluate the relationship between subject type and levels of SM 42:2 and SM 42:3. The results showed that there was a strong correlation with Kendall’s tau-b correlation coefficients of −0.515 and −0.493, respectively, which is significant at the 0.01 level (two-tailed test). Detailed data are shown in Table S1. Moving forward, it was assumed that SM 42:2 and SM 42:3 could be used as potential biomarkers and useful indicators in disease progression monitoring, as their level decreases with disease progression.

### 3.3. Characteristic and Biological Significance of Lipid Metabolism in Laryngeal Cancer

The investigation of differentially observed lipids screened in this study focused on phosphatidylcholine (PC), lysophosphatidylcholine (LPC), and sphingomyelin (SM) metabolism. Most of the differentially observed lipids exhibited varying degrees of down-regulation and reprogramming in the cancer population (Table S2). As evidenced by the number of differentially observed metabolites, the differences in lipid metabolism between HC and LBT were small; however, the LC group showed significant changes compared to other treatment groups.

**Figure 5:**
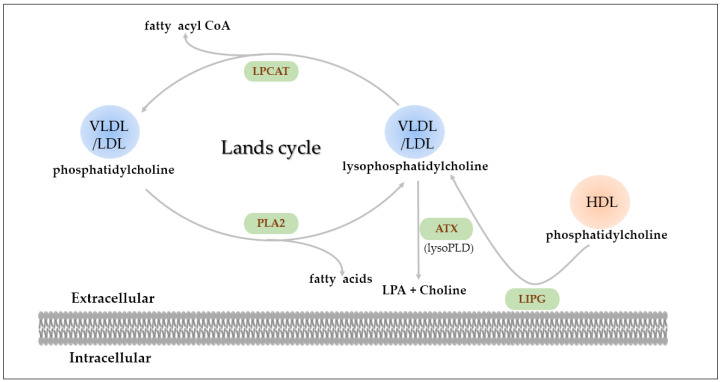
Degradation of phosphatidylcholine and lysophosphatidylcholine with involved key enzymes (VLDL, very low-density lipoprotein; LDL, low-density lipoprotein; HDL, high-density lipoprotein; LPA, lysophosphatidic acid; PLA2, phospholipase A2; LPCAT, lysophosphatidylcholine acyltransferase; ATX, autotoxin; LIPG, endothelial lipase).

Comparing serum lipids of LC to HC and LBT groups, 22 PC species and 17 LPC species were significantly different in the LC group (Figure 3). While a portion of the measured serum lipids are released from cells, the majority are generated during metabolic circulation by key serum enzymes involved in lipid–metabolic pathways (Figure 5).

Human lipoproteins, including very low-density lipoprotein (VLDL), low-density lipoprotein (LDL), and high-density lipoprotein (HDL), contain PC and LPC. The Lands cycle is a circulation mechanism for the synthesis and degradation of PC and LPC [14]. Phospholipase A2 (PLA2) hydrolyzes PC and is involved in the generation of LPC and other fatty acids [15]. It can also produce inflammatory mediators which promote the development of various cancers [16], and has been confirmed to be overexpressed in colorectal [17], breast [18], and lung cancers [19]. Therefore, the observation of significantly lower levels of most PC species in serum samples from LC patients could be taken as a proxy for PLA2 overexpression in the development of laryngeal cancer. Endothelial lipase (LIPG) primarily functions as a phospholipase with minor triglyceride lipase activity [20]. LIPG hydrolyzes extracellular HDL phosphatidylcholine, and releases lipid precursors including extracellular LPC species and fatty acids [20,21]. Studies have confirmed that LIPG is up-regulated in breast cancer cells and human colorectal adenocarcinoma cells [22,23]. Thus, the lower level of serum PC species in LC patients may be related to the high expression of LIPG.

In addition, some special lipids of interest should be noted. Ether phospholipids, such as PC O-34:2 and PC O-34:3, are a special class of phospholipid molecules that have been reported to be involved in membrane composition, cell differentiation, and signaling [24]. However, there has been less research on the physiological significance of ether phospholipids undertaken in tumors. Very-long-chain polyunsaturated phospholipids were commonly observed differential lipid classes. For example, PC 38:5 and PC 40:5 were differentially observed between HC and LC groups. A decreasing concentration has been reported in many polyunsaturated phospholipids within hepatocellular carcinoma, and this trend is consistent with the findings of the present study in laryngeal cancer [25]. Polyunsaturated phospholipids are extremely abundant in specific organelles (e.g., synaptic vesicles), and they may contribute to lipid raft formation, biofilm synthesis, and support of endocytosis [26,27].

LPC can be produced via catalysis by PLA2, and can also be converted back to PC by lysophosphatidylcholine acyltransferase (LPCAT) in the presence of acyl-CoA [28]. The direct degradation of LPC to lysophosphatidic acid (LPA) is catalyzed by autotoxin (ATX), a type of phospholipase D [14] which induces cell growth and triggers inflammation [29]. LPCAT promotes the down-regulation of LPC levels and its overexpression is associated with cancer, as demonstrated in oral squamous cell carcinoma and gastric cancer [30,31]. LPC16:0 and LPC18:0 were the two most abundant LPC species in serum, and their down-regulation would trigger a global decrease in total LPC levels. The reduction in LPC16:0 content has been confirmed in breast, ovarian, and colorectal cancers [32,33,34]. The level of LPC18:0 was inversely correlated with the risk of breast, prostate, and colorectal cancers, as well as melanoma [35,36]. In the present study, it was observed that LPC16:0 and LPC18:0 were present in lower levels in LC compared to the other two groups. The lower levels of the LPC species may reflect the increased metabolic rate of patients, which promotes tumor invasion, metastasis, and prognosis.

Furthermore, 15 SM species showed significant differences in serum of LC compared to HC and LBT groups (Figure 3). SM species are synthesized by sphingomyelin synthase, and can be converted, under the action of different enzymes, into ceramides, lysosphingolipids, glycosphingolipids, and sphingosines [37]. SM species are the basic components of lipid rafts and are involved in functionalizing transmembrane proteins [38]. In addition, SM can regulate processes, including inflammatory signaling, cell death, proliferation, and pain [39]. SM 42:2 and SM 42:3 belong to the class of long-chain sphingolipids. SM 42:2 was found to be reduced in lipid membranes of fibroblasts isolated from patients with Parkinson’s disease with the L444P GBA mutation [40], which was also present in lower levels in the LC group in this study. CD1a protein binds endogenous lipids and preferentially captures sphingolipids, especially SM 42:2, to block antigen receptor binding and impair T-cell responses [41]. In the progression of laryngeal cancer, it was assumed that SM42:2 may also play a role as a CD1a binding blocker, thereby weakening the immune response and promoting cancer metastasis.

This study has demonstrated the importance of lipid metabolism regulation in laryngeal cancer. However, our study is limited by its sample size and the screened lipid biomarkers are only used as potential diagnostic references. A blind sampling process for potential patients to be diagnosed should be carried out in future studies to examine the reliability of these biomarkers. An independent cohort with a larger number of samples could also be used to validate these lipid biomarkers.

## 4. Conclusions

In this study, we performed lipid extraction and completed UHPLC-QTOF-MS-based lipidomics analysis of serum from LC, LBT, and HC groups. A total of 57 lipids with significant differences were screened, and metabolic signatures that were mainly focused on PC, LPC, and SM metabolism were uncovered. The concentrations of SM 42:2 and SM 42:3 were correlated with disease progression, which points to their potential use as biomarkers for the diagnosis of laryngeal cancer. In this study, the changes in the regulation of the lipid metabolic network in laryngeal cancer using lipidomics were elucidated, and potential new diagnostic biomarkers of laryngeal cancer were found.

## Figures and Tables

**Table 1:** Baseline clinical pathological features of enrolled subjects.

Baseline Clinical Features of Enrolled Patients and Controls			
	HC	LC	LBT
Age (average, range)	51.9, 40–65	51.9, 42–80	51.8, 39–66
Sex (Male/Female)	5/5	6/4	6/4
Cases	10	10	10

P-value (age) = 0.611. The baseline clinical pathological features of enrolled volunteers were analyzed by one-way analysis of variance (ANOVA), and their ages conformed to a normal distribution and met the requirement of a chi-square test. HC, healthy crowds; LC, laryngeal cancer patients; LBT, laryngeal benign tumor patients.

## Data Availability

All the data that support the findings of this study are available within the manuscript, Supplementary Materials, or from the corresponding authors upon request.

## Supplementary Materials

The following supporting information can be downloaded at: https://www.mdpi.com/article/10.3390/metabo12111087/s1, Table S1: Bivariate correlation analysis of subjects type and SM 42:2, SM42:3, Table S2: Significant different lipids in serum of healthy crowds, laryngeal cancer crowd, and laryngeal benign tumor crowd, Table S3: Qualitative information on 57 differential lipids.
