# Supplementary material for: Non-Targeted Serum Lipidomics Analysis and Potential Biomarkers of Laryngeal Cancer Based on UHPLC-QTOF-MS
Source: Metabolites. 2022 Nov 9;12(11):1087. doi: 10.3390/metabo12111087 (PMC9695307; doi:10.3390/metabo12111087)
Supplement: Supplementary file 1 [file metabolites-12-01087-s001.zip › metabolites-1988330-supplementary.pdf]

Supporting information

Non-targeted serum lipidomics analysis and potential biomarkers of laryngeal cancer based on UHPLC-QTOF-MS

Haoyue Wang [1,2,3], Yanbo Luo [3], Huan Chen [3], Hongwei Hou [3], Qingyuan Hu [3,*], Min Ji [1,*]

[1] Institute of Optics and Fine Mechanics, Hefei Institutes of Physical Science, Chinese Academy of Sciences, Hefei 230031, China
[2] University of Science and Technology of China, Hefei 230026, China
[3] Key Laboratory of Tobacco Biological Effects, China National Tobacco Quality Supervision & Test Center, Zhengzhou 45000l, China
[*] Correspondence: huqy@tbescience.com (Q.H.); jimin@aiofm.ac.cn (M.J.)

Table of contents

- **Table S1.** Bivariate correlation analysis of subjects type and SM 42:2, SM42:3.
- **Table S2.** Qualitative information of 57 differential lipids.
- **Table S3.** Significant different lipids in serum of healthy crowd, laryngeal cancer crowd and laryngeal benign tumor crowd.

Table S1. Bivariate correlation analysis of subjects type and SM 42:2, SM42:3.

			subjects type	SM 42:2
Kendall's tau_b	subjects type	Correlation Coefficient	1.000	-.515**
		Sig. (2-tailed)		0.000
		N	30	30
	SM 42:2	Correlation Coefficient	-.515**	1.000
		Sig. (2-tailed)	0.000	
		N	30	30

**. Correlation is significant at the 0.01 level(2-tailed).

			subjects type	SM 42:3
Kendall's tau_b	subjects type	Correlation Coefficient	1.000	-0.493**
		Sig. (2-tailed)		0.001
		N	30	30
	SM 42:2	Correlation Coefficient	-.493**	1.000
		Sig. (2-tailed)	0.001	
		N	30	30

**. Correlation is significant at the 0.01 level(2-tailed).

Table S2. Qualitative information of 57 differential lipids

Code	Compound	Retention Time（min）	m/z	M	mSigma	Δm/z [ppm]	Δm/z [mDa]	MS/MS score	Aducct	Molecular Formula
1	SM 42:2;2O	14.44	813.6834	812.6760	9.7	-1.603	-1.304	840.7	[M+H]+, [M+Na]+	C47H93N2O6P
2	SM 42:3;2O	12.34	811.6675	810.6602	5.8	-1.715	-1.392	758.5	[M+H]+, [M+Na]+	C47H91N2O6P
3	LPC 14:0	0.91	468.3077	467.3004	25.5	-1.597	-0.748	801.9	[M+H]+, [M+K]+, [M+Na]+	C22H46NO7P
4	LPC 16:0-SN1	1.13	496.3392	495.3318	10.5	-1.178	-0.585	954.2	[M+H]+, [M+Na]+	C24H50NO7P
5	LPC 16:1-SN1	0.95	494.3234	493.3161	5.1	-1.519	-0.751	920.2	[M+H]+	C24H48NO7P
6	LPC 18:0	1.67	524.3706	523.3631	7.3	-1.332	-0.699	736.6	[M+H]+, [M+Na]+, [M-H2O+H]+, [M+K]+	C26H54NO7P
7	LPC 18:1-SN1	1.24	522.3548	521.3474	11.6	-1.215	-0.635	935.7	[M+H]+, [M+Na]+	C26H52NO7P
8	LPC O-16:1	1.32	480.3439	479.3367	2.4	-1.806	-0.867	801.1	[M+H]+, [M+Na]+	C24H50NO6P
9	LPC O-18:1	1.45	508.3752	507.3680	31.8	-1.797	-0.914	986.3	[M+H]+, [M+Na]+	C26H54NO6P
10	LPC O-18:0	2.01	510.3909	509.3836	6	-1.782	-0.909	989.3	[M+H]+	C26H56NO6P

11	LPC 18:2-SN1	1	520.3393	519.3319	11.2	-0.965	-0.502	946.5	[M+H]+, [M+Na]+, [M-H2O+H]+	C26H50NO7P
12	LPC 18:3-SN1	0.88	518.3232	517.3160	6.6	-1.872	-0.97	939	[M+H]+, [M+K]+	C26H48NO7P
13	PC 38:5	7.6	808.5842	807.5767	2.5	-1.639	-1.325	750.9	[M+H]+, [M+Na]+	C46H82NO8P
14	PC 15:0_18:2	6.82	744.5526	743.5454	10.3	-1.538	-1.145	988.3	[M+H]+	C41H78NO8P
15	PC 16:0_18:3	6.51	756.5527	755.5454	9.3	-1.517	-1.148	986.9	[M+H]+	C42H78NO8P
16	PC 16:0_20:5	6.32	780.5526	779.5453	10.6	-1.488	-1.161	992.2	[M+H]+, [M+Na]+	C44H78NO8P
17	PC 16:1_16:1	6	730.5370	729.5295	9.2	-1.605	-1.173	992.7	[M+H]+, [M+K]+, [M+Na]+	C40H76NO8P
18	PC 17:1_17:1	8.1	758.5683	757.5610	4.1	-1.493	-1.132	992.2	[M+H]+	C42H80NO8P
19	PC 17:2_17:2	5.79	754.5367	753.5303	3.1	-1.934	-1.46	993.4	[M+H]+, [M+Na]+	C42H76NO8P
20	PC 18:1_18:1	9.82	786.5997	785.5924	16.2	-1.294	-1.018	989	[M+H]+	C44H84NO8P
21	PC 18:2_18:2	6.55	782.5686	781.5613	14.7	-1.072	-0.839	988.4	[M+H]+	C44H80NO8P
22	PC 18:2_20:4	6.31	806.5680	805.5607	16.3	-1.778	-1.434	989.1	[M+H]+, [M+Na]+	C46H80NO8P
23	PC 36:1	12.41	788.6154	787.6080	8.9	-1.563	-1.232	729.6	[M+H]+, [M+K]+, [M+Na]+	C44H86NO8P
24	PC O-34:2	10.67	744.5889	743.5816	4.3	-1.742	-1.297	994.2	[M+H]+	C42H82NO7P
25	PC O-34:3	8.69	742.5735	741.5662	10.8	-1.405	-1.044	993	[M+H]+	C42H80NO7P

26	SM 16:1;2O/23:0	13.69	773.6517	772.6444	12.7	-1.854	-1.434	997	[M+H]+	C44H89N2O6P
27	SM 18:1;2O/22:0	14.48	787.6678	786.6604	15	-1.242	-0.978	992.6	[M+H]+, [M+Na]+	C45H91N2O6P
28	SM 18:2;2O/16:0	5.55	701.5583	700.5511	15.7	-1.283	-0.9	991	[M+H]+	C39H77N2O6P
29	SM 18:2;2O/20:0	9.47	757.6205	756.6131	1.2	-1.73	-1.311	996.6	[M+H]+, [M+Na]+	C43H85N2O6P
30	SM 18:2;2O/22:0	12.44	785.6519	784.6446	11.4	-1.517	-1.192	997.4	[M+H]+, [M+Na]+	C45H89N2O6P
31	SM 18:2;2O/23:0	14.02	799.6674	798.6601	6.7	-1.792	-1.433	994.8	[M+H]+, [M+Na]+	C46H91N2O6P
32	SM 18:2;2O/24:0	14.64	813.6833	812.6759	16	-1.356	-1.103	993.2	[M+H]+, [M+Na]+	C47H93N2O6P
33	SM 41:1;2O	14.91	801.6832	800.6759	2.6	-1.717	-1.377	847.2	[M+H]+, [M+Na]+	C46H93N2O6P
34	PC O-44:6	14.41	876.6822	875.6748	6.1	-2.111	-1.851	996.7	[M+H]+, [M+Na]+	C52H94NO7P
35	LPC 15:0-SN1	1.03	482.3233	481.3162	1.5	-1.722	-0.831	916.1	[M+H]+, [M+Na]+	C23H48NO7P
36	LPC 17:0-SN1	1.39	510.3547	509.3474	1.6	-1.444	-0.737	917.9	[M+H]+, [M+Na]+	C25H52NO7P
37	LPC 20:3-SN1	1.09	546.3544	545.3471	6.9	-2.015	-1.101	953.3	[M+H]+, [M+K]+	C28H52NO7P
38	LPC 20:4-SN1	0.97	544.3389	543.3318	12.1	-1.509	-0.821	936.6	[M+H]+, [M-H2O+H]+, [M+K]+	C28H50NO7P
39	LPC O-16:0	1.38	482.3594	481.3521	3	-2.327	-1.122	967.2	[M+H]+	C24H52NO6P

40	PC 16:0_18:1	9.51	760.5846	759.5773	29.2	-0.705	-0.536	983.9	[M+H]+	C42H82NO8P
41	PC 38:5	7.6	808.5842	807.5767	2.5	-1.639	-1.325	750.9	[M+H]+,<br>[M+Na]+	C46H82NO8P
42	PC 40:5	9.87	836.6150	835.6076	14.6	-1.996	-1.67	681.1	[M+H]+,<br>[M+Na]+	C48H86NO8P
43	SM 16:1;2O/22:0	11.93	759.6362	758.6293	7.8	-1.601	-1.216	995.7	[M+H]+,<br>[M+K]+,<br>[M+Na]+	C43H87N2O6P
44	SM 18:1;2O/16:0	6.91	703.5742	702.5670	9.9	-0.883	-0.621	991.2	[M+H]+	C39H79N2O6P
45	SM 18:1;2O/18:0	9.07	731.6051	730.5978	9.3	-1.444	-1.057	993.2	[M+H]+,<br>[M+Na]+	C41H83N2O6P
46	SM 18:2;2O/18:0	7.23	729.5894	728.5821	6.5	-1.504	-1.098	994	[M+H]+,<br>[M+Na]+	C41H81N2O6P
47	PC 17:0_18:2	8.54	772.5840	771.5767	2.8	-1.362	-1.052	991.1	[M+H]+	C43H82NO8P
48	PC 17:0_20:4	8.54	796.5839	795.5766	4.9	-1.545	-1.231	991.3	[M+H]+	C45H82NO8P
49	PC O-38:4	10.54	796.6194	795.6125	3.8	-2.625	-2.091	994.9	[M+H]+,<br>[M+Na]+	C46H86NO7P
50	LPC 44:5	15.06	878.6980	877.6907	50.6	-2.146	-1.886	572.7	[M+H]+,<br>[M+Na]+	C52H96NO7P
51	PC 16:0_20:3	8.6	784.5839	783.5766	9.5	-1.494	-1.172	990.3	[M+H]+	C44H82NO8P
52	PC 18:0_20:3	11.22	812.6150	811.6077	8.6	-1.779	-1.446	991.5	[M+H]+	C46H86NO8P
53	PC 30:0	7.07	706.5366	705.5295	81	-1.826	-1.29	712.9	[M+H]+,<br>[M+Na]+	C38H76NO8P
54	PC O-36:4	8.7	768.5892	767.5830	13.5	-1.323	-1.017	990.9	[M+H]+,<br>[M+K]+	C44H82NO7P
55	PC O-40:4	13.7	824.6510	823.6437	6.1	-2.143	-1.767	995.6	[M+H]+	C48H90NO7P

56	PC O-42:5	14.25	850.6669	849.6590	2.9	-1.863	-1.584	996.6	[M+H]+, [M+Na]+	C50H92NO7P
57	SM 29:0;2O/5:0	7.64	705.5894	704.5821	4.4	-1.56	-1.101	995.8	[M+H]+	C39H81N2O6P

Table S3. Significant different lipids in serum of healthy crowd, laryngeal cancer crowd and laryngeal benign tumor crowd.

Code	Compounds	P	VIP	Trends	Group
1	SM 42:2_2O	***	5.32	down	LC V.S. HC
		*	2.26	down	LBT V.S. HC
		***	5.53	down	LC V.S. LBT
2	SM 42:3_2O	***	2.37	down	LC V.S. HC
		**	1.52	down	LBT V.S. HC
		**	1.94	down	LC V.S. LBT
3	LPC 14:0-SN1	***	1.93	down	LC V.S. HC
		**	1.50	down	LBT V.S. HC
4	LPC 16:0-SN1	***	7.01	down	LC V.S. HC
		***	4.18	down	LBT V.S. HC
5	LPC 16:1-SN1	***	2.03	down	LC V.S. HC
		***	2.19	down	LBT V.S. HC
6	LPC 18:0-SN1	***	8.78	down	LC V.S. HC
		***	7.94	down	LBT V.S. HC
7	LPC 18:1	**	4.81	up	LC V.S. HC
		*	3.98	down	LBT V.S. HC
8	LPC O-16:1	***	1.26	down	LC V.S. HC
		***	1.21	down	LBT V.S. HC
9	LPC O-18:1	***	1.01	down	LC V.S. HC
		***	1.11	down	LBT V.S. HC
10	PC O-18:0	***	2.94	down	LC V.S. HC
		***	3.07	down	LBT V.S. HC
11	LPC 18:2-SN1	*	5.38	down	LC V.S. HC
		*	6.67	down	LC V.S. LBT
12	LPC 18:3-SN1	***	1.20	down	LC V.S. HC
		**	1.12	down	LC V.S. LBT
13	LPC 38:5	*	1.69	down	LC V.S. HC
		*	2.03	down	LC V.S. LBT
14	PC 15:0_18:2	*	1.39	down	LC V.S. HC
		**	2.25	down	LC V.S. LBT
15	PC 16:0_18:3	***	1.89	down	LC V.S. HC
		***	2.89	down	LC V.S. LBT
16	PC 16:0_20:5	***	1.29	down	LC V.S. HC
		***	1.40	down	LC V.S. LBT
17	PC 16:1_16:1	**	2.09	down	LC V.S. HC
		**	2.18	down	LC V.S. LBT
18	PC 17:1_17:1	***	2.00	down	LC V.S. HC
		***	2.28	down	LC V.S. LBT

19	PC 17:2_17:2	**	1.05	down	LC V.S. HC
		**	1.28	down	LC V.S. LBT
20	PC 18:1_18:1	*	5.38	down	LC V.S. HC
		*	6.05	down	LC V.S. LBT
21	PC 18:2_18:2	*	3.63	down	LC V.S. HC
		**	6.23	down	LC V.S. LBT
22	PC 18:2_20:4	***	2.54	down	LC V.S. HC
		***	3.45	down	LC V.S. LBT
23	PC 36:1	**	3.01	up	LC V.S. HC
		*	3.26	up	LC V.S. LBT
24	PC O-34:2	*	1.07	down	LC V.S. HC
		*	1.56	down	LC V.S. LBT
25	PC O-34:3	***	1.99	down	LC V.S. HC
		**	2.12	down	LC V.S. LBT
26	SM 16:1_2O_23:0	***	1.06	down	LC V.S. HC
		***	1.21	down	LC V.S. LBT
27	SM 18:1_2O_22:0	**	3.84	down	LC V.S. HC
		**	4.18	down	LC V.S. LBT
28	SM 18:2_2O_16:0	**	2.63	down	LC V.S. HC
		*	2.58	down	LC V.S. LBT
29	SM 18:2_2O_20:0	***	1.18	down	LC V.S. HC
		**	1.19	down	LC V.S. LBT
30	SM 18:2_2O_22:0	**	1.73	down	LC V.S. HC
		*	2.07	down	LC V.S. LBT
31	SM 18:2_2O_23:0	***	1.99	up	LC V.S. HC
		***	2.46	up	LC V.S. LBT
32	SM 18:2_2O_24:0	***	3.03	down	LC V.S. HC
		**	3.68	down	LC V.S. LBT
33	SM 41:1_2O	**	2.53	up	LC V.S. HC
		**	2.83	up	LC V.S. LBT
34	PC O-44:6	**	1.16	down	LBT V.S. HC
		***	1.83	up	LC V.S. LBT
35	LPC 15:0-SN1	***	1.02	down	LC V.S. HC
36	LPC 17:0-SN1	*	1.04	down	LC V.S. HC
37	LPC 20:3-SN1	*	1.12	down	LC V.S. HC
38	LPC 20:4-SN1	**	2.72	down	LC V.S. HC
39	LPC O-16:0	***	1.21	down	LC V.S. HC
40	PC 16:0_18:1	*	5.69	down	LC V.S. HC
41	PC 38:5	**	3.19	up	LC V.S. HC
42	PC 40:5	**	1.48	down	LC V.S. HC
43	SM 16:1_2O_22:0	*	1.19	down	LC V.S. HC
44	SM 18:1_2O_16:0	**	4.59	down	LC V.S. HC
45	SM 18:1_2O_18:0	**	1.70	down	LC V.S. HC
46	SM 18:2_2O_18:0	**	1.58	down	LC V.S. HC

47	LPC 44:5	**	1.50	down	LC V.S. LBT
48	PC 16:0_20:3	*	1.70	down	LC V.S. LBT
49	PC 18:0_20:3	*	1.09	down	LC V.S. LBT
50	PC 30:0	*	1.41	down	LC V.S. LBT
51	PC O-36:4	*	2.30	down	LC V.S. LBT
52	PC O-40:4	***	1.19	down	LC V.S. LBT
53	PC O-42:5	***	1.36	down	LC V.S. LBT
54	SM 29:0_2O_5:0	**	1.03	down	LC V.S. LBT
55	PC 17:0_18:2	**	1.95	up	LBT V.S. HC
56	PC 17:0_20:4	**	1.11	up	LBT V.S. HC
57	PC O-38:4	**	1.14	up	LBT V.S. HC

*P<0.05 **P<0.01 ***P<0.001
